# Supplementary material for: Metagenomic profiling and predictive modeling of the gut microbiome reveal signatures of gestational disease
Source: Microbiol Spectr. 2026 Mar 25;14(5):e03155-25. doi: 10.1128/spectrum.03155-25 (PMC13141881; doi:10.1128/spectrum.03155-25)
Supplement: Supplemental material — Fig. S1 to S12; Tables S1 and S2. [file spectrum.03155-25-s0001.pdf]

Supplementary Material

Supplementary Figure 1. Case/Control stratification of selected taxa

We justify the statistical analysis section of our Methods further by presenting the batch-effect normalization procedure results as NMDS and PCoA ordinations with samples colored by batch.

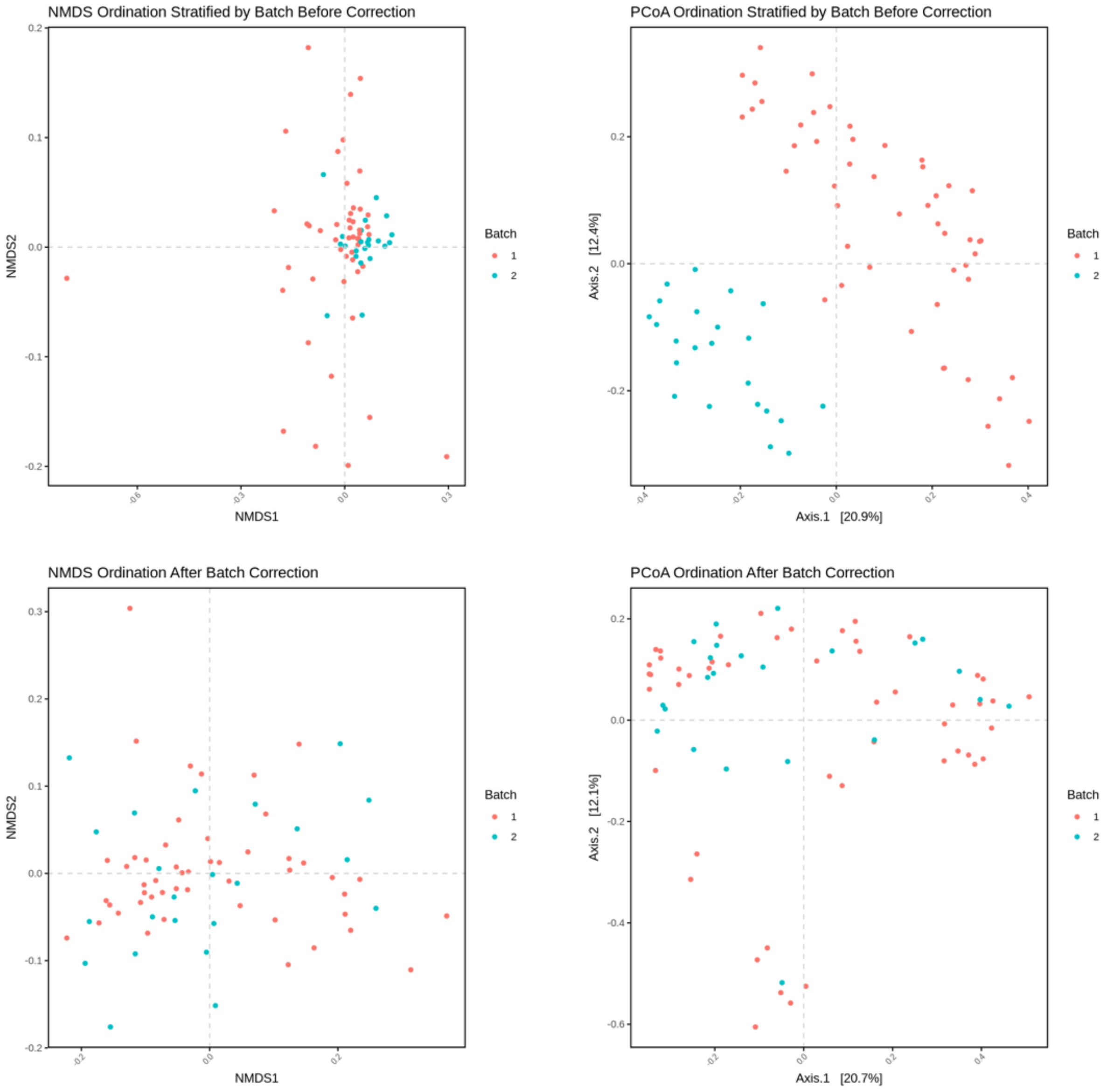

Supplementary Table 1. Comparison of clinical variables between case and control groups

We expand Table 1 of our Results by presenting a comprehensive comparison of maternal clinical, vital sign, and laboratory variables between the control (n=31) and case (n=42) groups. P-values are computed using independent two-sample t-tests for continuous variables and Fisher's exact tests for categorical variables. Group assignment was based on clinical diagnosis of gestational disease (GDM or hypertension), and those variables are not included here to avoid redundancy.

Table 1: Comparison of clinical variables between case and control groups.

**Variable**	**Test**	**p-value**
Preeclampsia During Labor	Fisher	**0.0018**
BMI (kg/m^2)	t-test	**0.0032**
Sex of Baby	Fisher	**0.0038**
LDL (mg/dL)	t-test	**0.0053**
Delivery EGA (weeks)	t-test	**0.0085**
Diastolic BP (mmHg)	t-test	**0.0118**
Systolic BP (mmHg)	t-test	**0.0136**
Birth Weight (g)	t-test	**0.0172**
Triglycerides (mg/dL)	t-test	**0.0214**
Total Cholesterol (mg/dL)	t-test	**0.0471**
Delivery Type	Fisher	0.0916
Stool Count	t-test	0.1770
Fructosamine (mmol/L)	t-test	0.2108
Maternal Age at Consent	t-test	0.2879
Sample Batch	Fisher	0.3119
Preeclampsia Postpartum	Fisher	0.3365
HDL (mg/dL)	t-test	0.4581
Consent EGA (weeks)	t-test	0.4904
Preterm Labor	Fisher	0.5070
Enroll GA from REDCap	t-test	0.5154
HbA1c (%)	t-test	0.5571
V1 EGA Biometric (weeks)	t-test	0.5699
Visit Timing	Fisher	0.8163
Glucose (mg/dL)	t-test	0.8271
Heart Rate (bpm)	t-test	0.8404
Race	Fisher	0.9353
Total Cholesterol (dup)	t-test	0.9950
Tobacco Use	Fisher	1.0000

Supplementary Figure 2. Case/Control stratification of selected taxa

This figure expands Figure 3A in the main text by displaying selected taxa known to be associated with GDM and GH in a boxplot stratified by cases and controls.

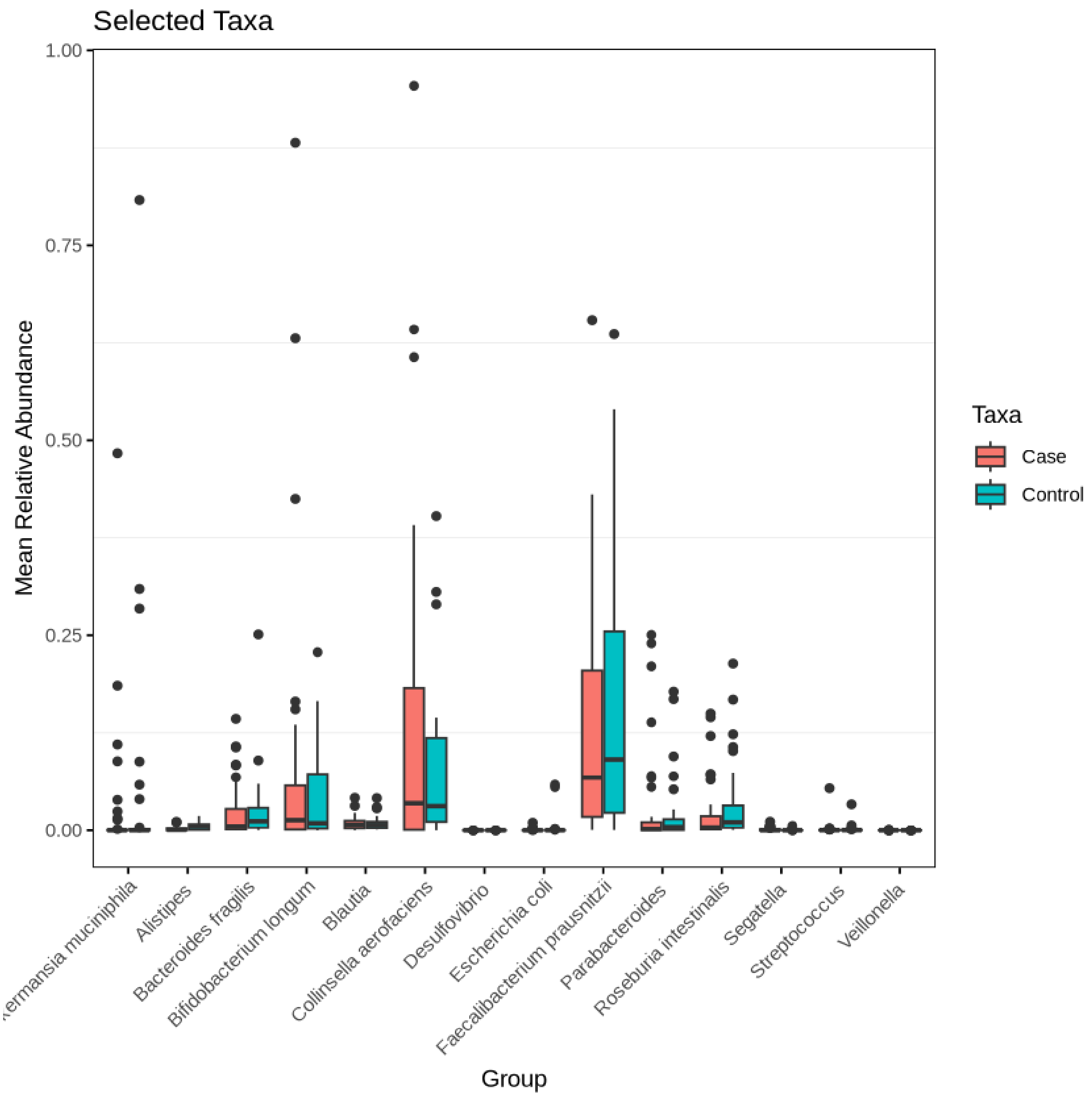

Supplementary Figure 3. F/B ratios of cases and controls

This figure displays the ratio of *Firmicutes* to *Bacteroidetes* as a standardized way of compared microbial consistencies between two groups. We take the raw abundances of relevant taxon within each phyla, Winsorize outliers, then calculate mean F/B per group. We also perform a Wilcoxon ranked sum test to establish significance in differences seen between groups.

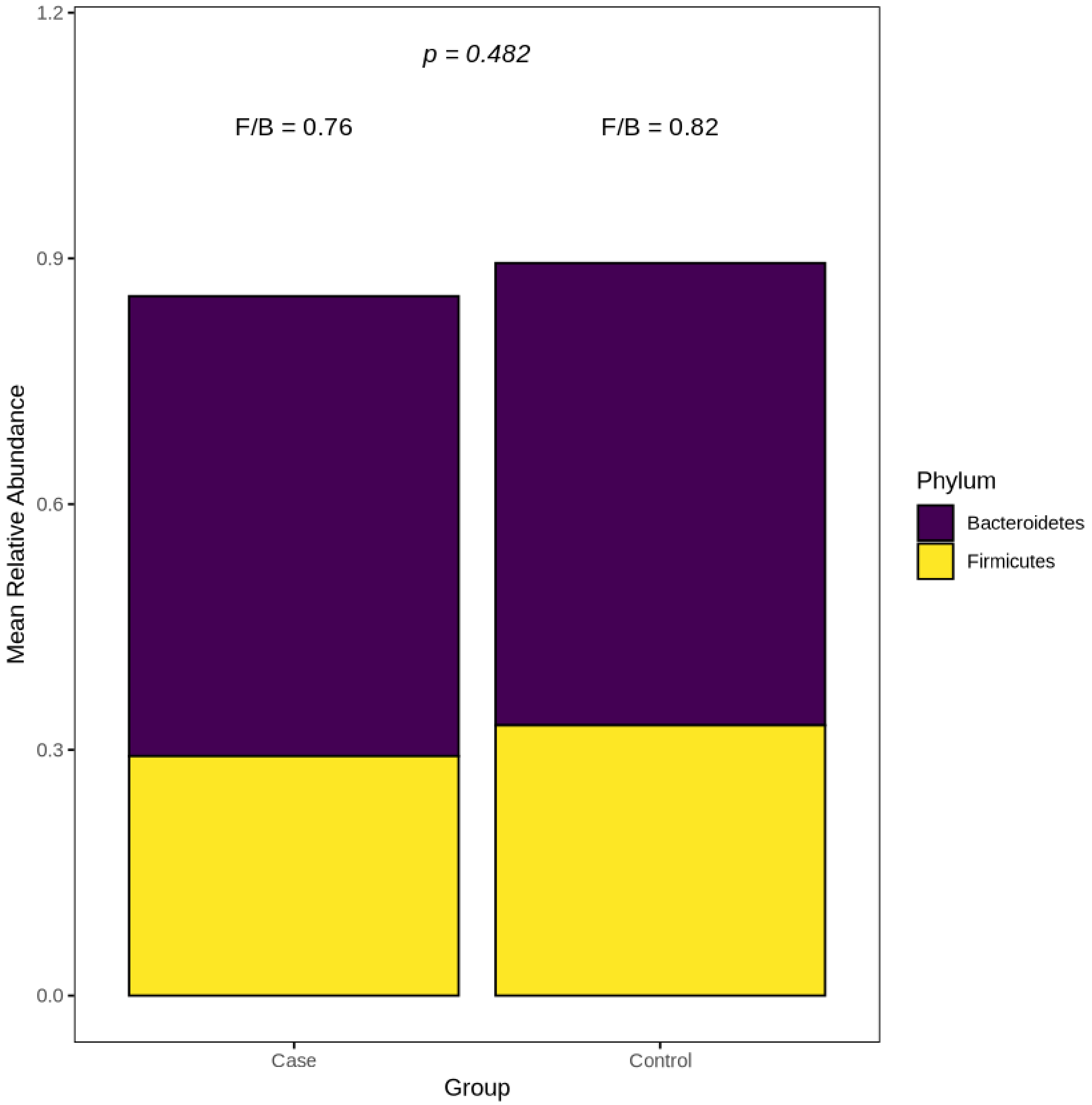

Supplementary Figure 4. Feature correlation with metadata variables

To evaluate the clinical relevance of differentially enriched microbial features, Spearman correlation analyses were performed between host clinical indicators and (A) differentially abundant bacterial taxa and (B) differentially enriched functional pathways. Correlation coefficients are shown for each feature–metadata pair. Associations marked with an asterisk (*) indicate nominal significance ($p < 0.05$), while those marked with a plus sign (+) indicate stronger significance ($p < 0.01$). These analyses provide exploratory evidence linking microbial taxonomic and functional variation with host metabolic and clinical parameters.

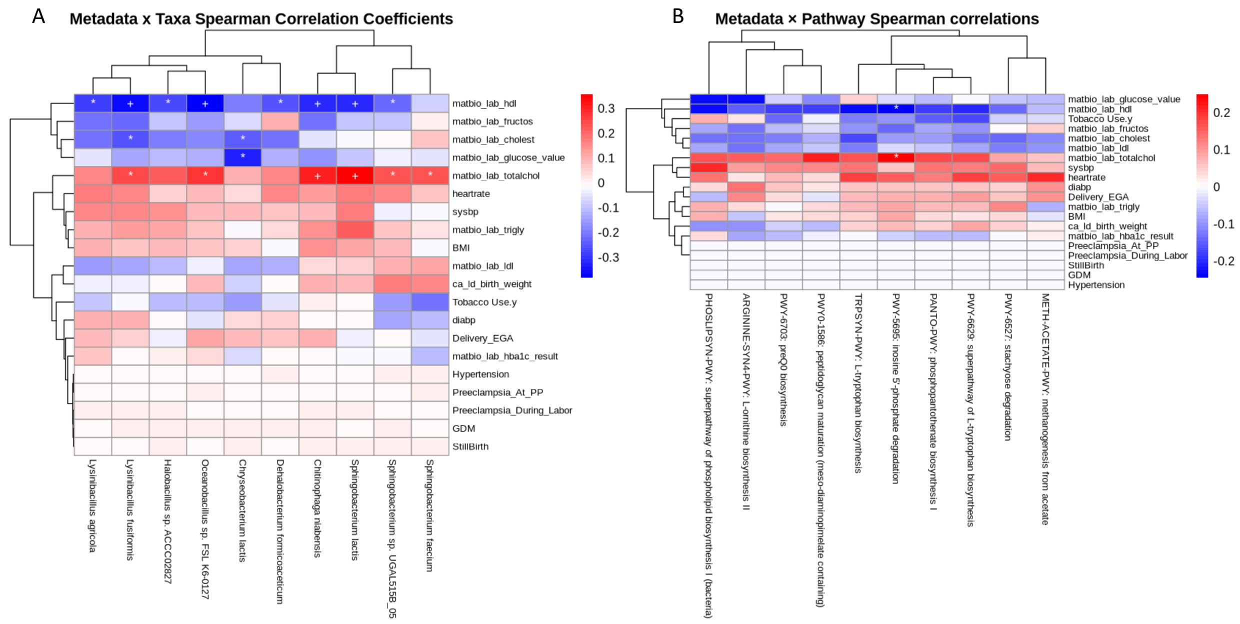

Supplementary Figure 5. Differential abundance analysis results

Both plots present differentially abundant taxa as a function of the log fold change. The volcano plot (A) colors taxa which have one full log fold change of abundance different between experimental groups to a significant degree (adjusted $p < 0.1$). The Mean-Abundance plot (B) presents the taxa which were differentially abundant to one full log fold difference between experimental groups and were above the mean of normalized counts for each taxa, but colored only if the taxa were also significantly differentially abundant as well.

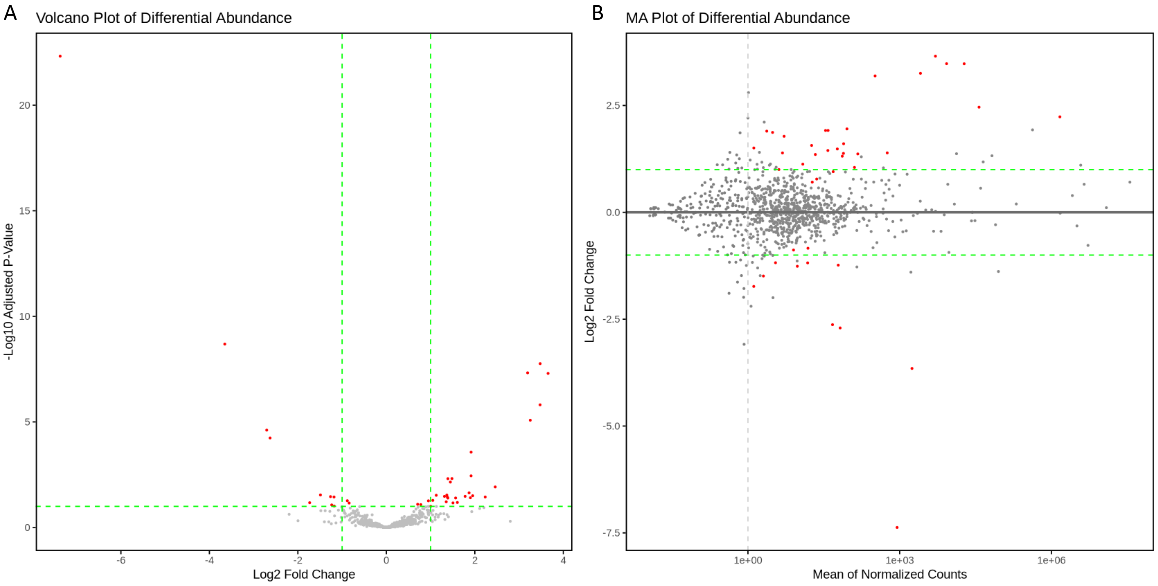

Supplementary Table 2. QC metrics of metagenomic sequencing data

Metagenomic sequencing generated a mean of 29.99 million raw reads per sample, with 29.49 million reads retained after quality control (mean retention: 98.13%). Sequencing quality was high across samples (mean Q30 = 0.92), and no samples were excluded based on sequencing depth (minimum threshold: 1,000 reads). No residual host genome sequences were detected because the sequencing facility provided data that had already been depleted of host DNA. To reduce noise from sparsely observed features, taxa and pathways were filtered using a prevalence threshold of ≥10% of samples. High sequencing depth and prevalence-based filtering improve the stability of taxonomic and functional profiling by minimizing the influence of low-quality reads and rare features.

Metric	Value
Raw reads per sample	29991645.89 ± 22128350.19 (1183758.00–85109094.00)
Post-QC reads per sample	29489371.62 ± 21859777.86 (1159156.00–84321070.00)
Read retention (%)	98.13 ± 0.34 (97.36–99.10)
Q30 rate	0.92 ± 0.01 (0.91–0.93)
Host DNA removal (%)	None present in samples
Microbial fraction	1.00 ± 0.00 (1.00–1.00)
Samples excluded (< 1000 reads)	0

Supplementary Figure 6. Technical flowchart

Overview of the metagenomic data processing, statistical analysis, and predictive modeling workflow. Stool samples were subjected to standardized shotgun metagenomic sequencing and processed using the MG-Pipe bioinformatics pipeline, followed by taxonomic and functional profiling, batch correction, differential abundance testing, and supervised machine learning analyses.

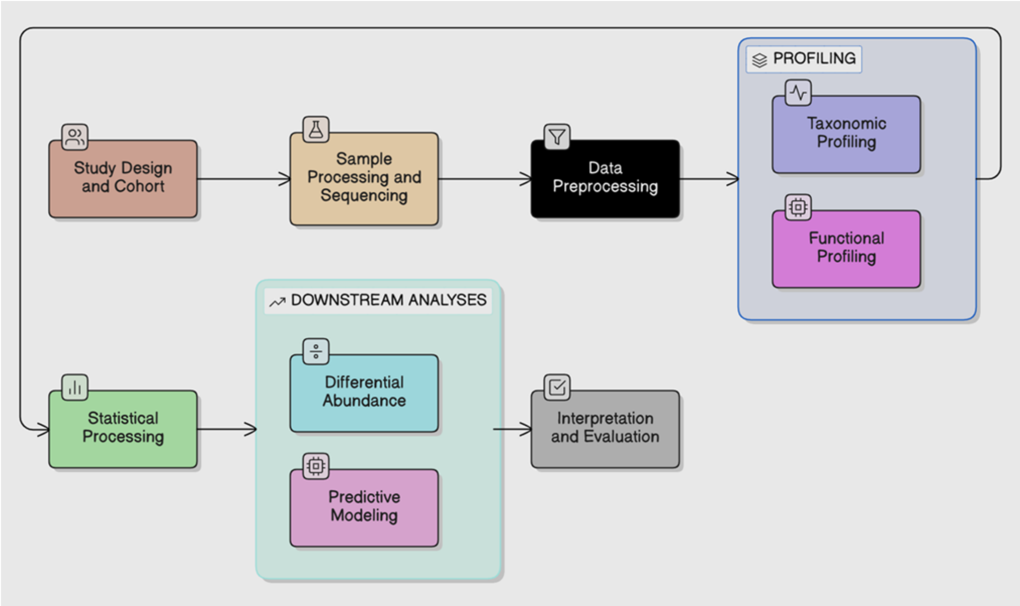

Supplementary Figure 7. Stratified alpha diversities

Presented in the figure as part of sensitivity analysis, we show that group specific microbial communities have low statistical power for differences between groups per condition. In panel A, patients with GDM versus healthy controls show no statistically or nominally significant community differences. The result is similar for panel B, which compares controls with GH patients.

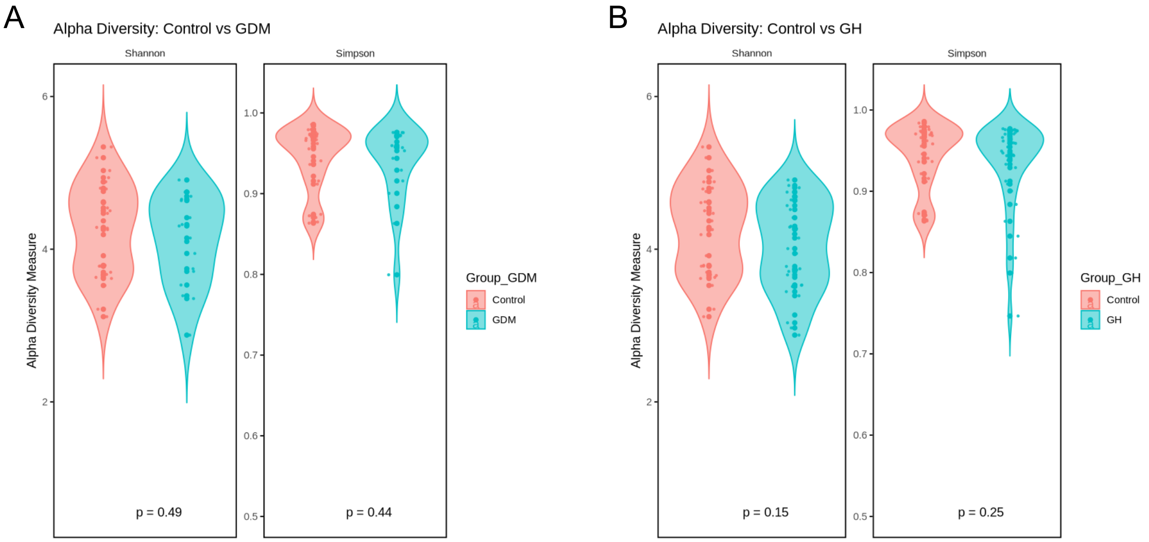

Supplementary Figure 8. Stratified beta diversity (Controls vs. GDM)

Beta diversity in GDM was assessed using Bray–Curtis dissimilarity and visualized by PCoA and NMDS ordinations. Group differences were tested using PERMANOVA, with 95% confidence ellipses shown; NMDS stress values are reported.

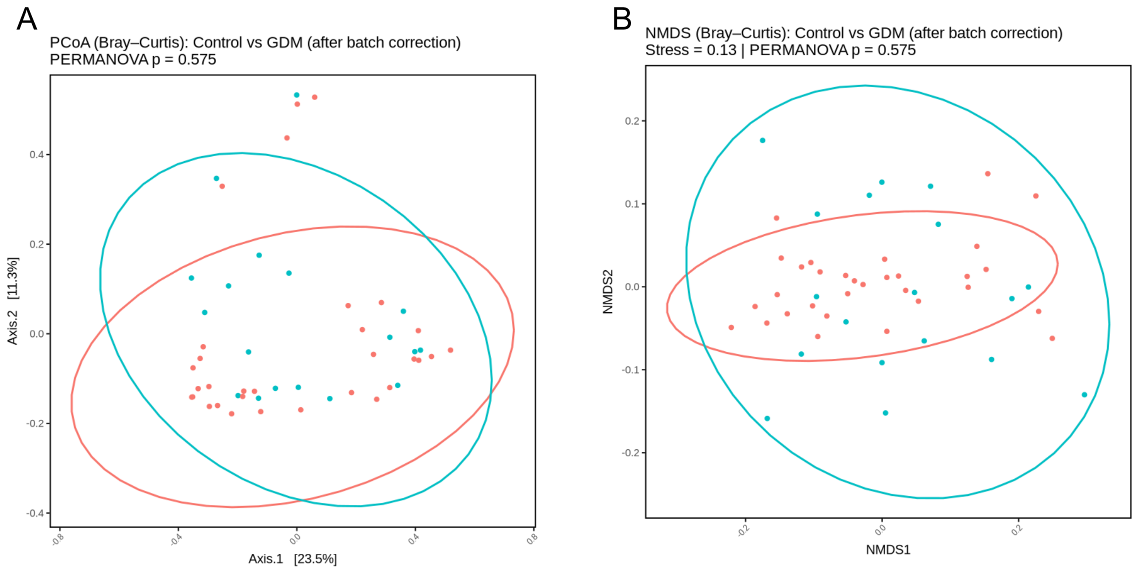

Supplementary Figure 9. Stratified beta diversity (Controls vs. GH)

Beta diversity in GDM was assessed using Bray–Curtis dissimilarity and visualized by PCoA and NMDS ordinations. Group differences were tested using PERMANOVA, with 95% confidence ellipses shown; NMDS stress values are reported.

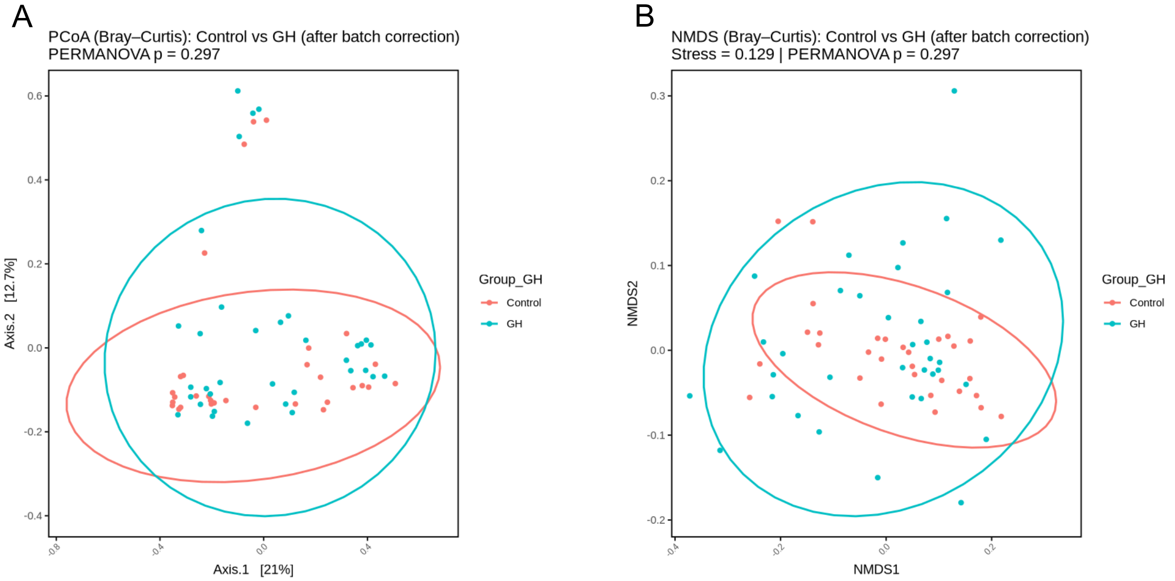

Supplementary Figure 10. Stratified differential abundance (Controls vs. GDM)

Differentially abundant taxa in GDM are shown as a function of log fold change. The volcano plot (A) highlights taxa with an absolute log fold change greater than or equal to 1 and adjusted $p < 0.1$. The mean–abundance plot (B) displays taxa meeting the same fold-change threshold and exceeding the mean normalized count, with color indicating statistically significant differential abundance.

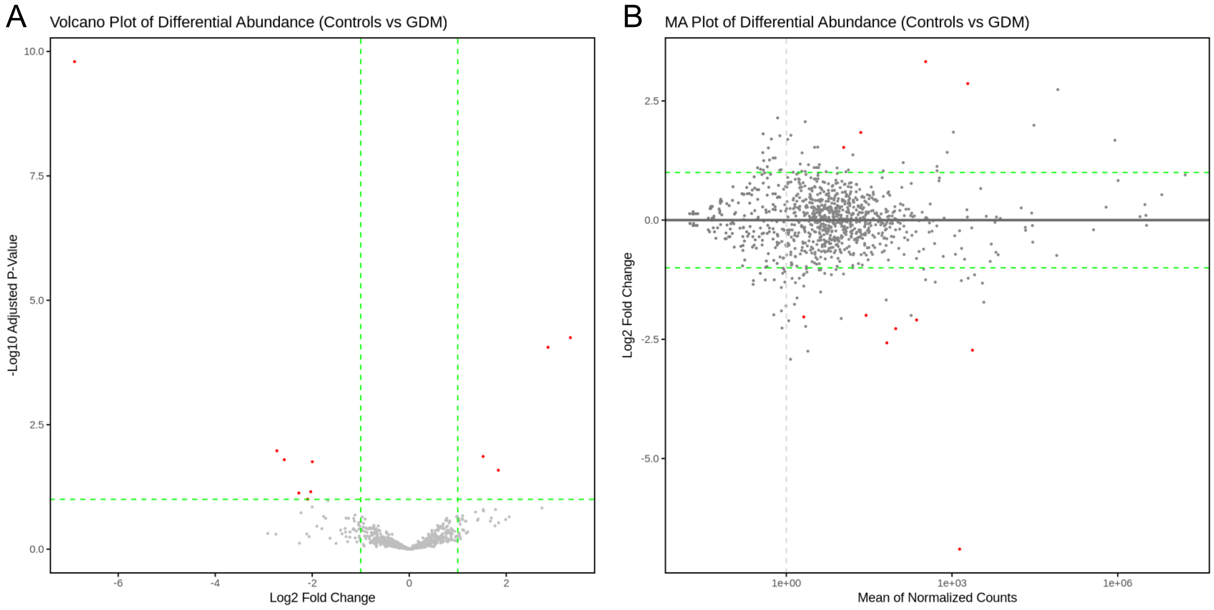

Supplementary Figure 11. Stratified differential abundance (Controls vs. GH)

Differentially abundant taxa in GH are shown as a function of log fold change. The volcano plot (A) highlights taxa with an absolute log fold change more than or equal to 1 and adjusted p < 0.1. The mean–abundance plot (B) displays taxa meeting the same fold-change threshold and exceeding the mean normalized count, with color indicating statistically significant differential abundance.

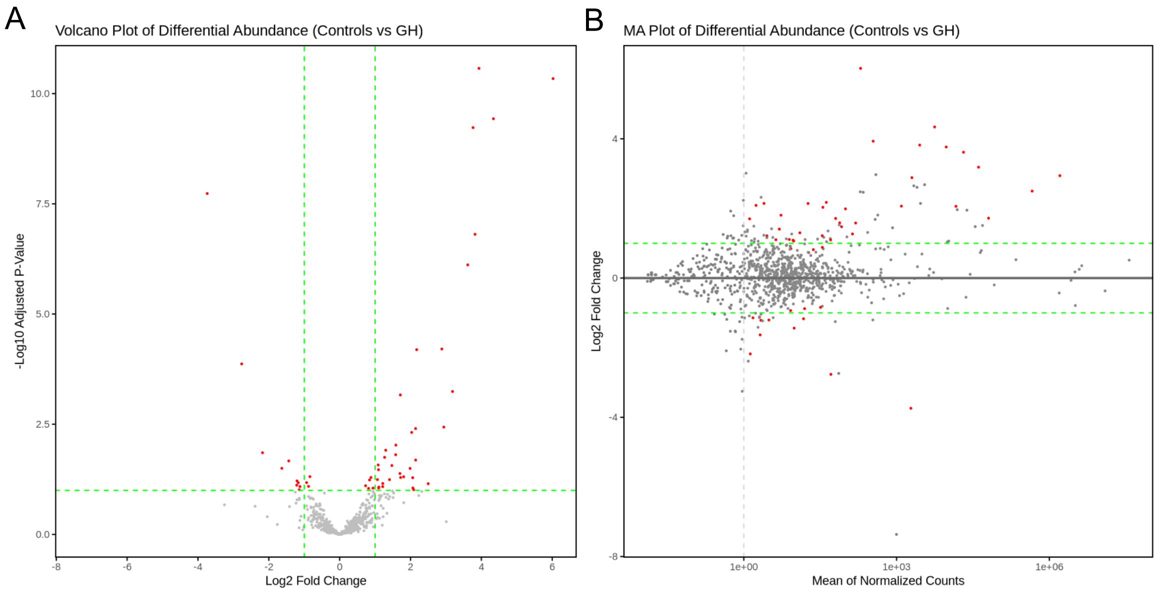

Supplementary Figure 12. Microbial overlap analysis

Using a significance threshold of $p < 0.05$, we identify the taxa which are significantly differentially abundant in GDM and GH cohorts independently relative to healthy controls. Of these, 5 microbial species were identified as as differentially abundant and statistically significant taxa in both disease cohorts. These species were *Dehalobacterium formicoaceticum, Lysinibacillus agricola, Paenibacillus sp. FSL E2-0178, Sphingobacterium faecium* and *Chitinophaga niabensis*. These taxa reinforce the results seen in our combined cohort in the main text, however, *Dehalobacterium formicoaceticum, Paenibacillus sp. FSL E2-0178* and *Chitinophaga niabensis* are not highly abundant and are likely identified due to the small sample size when separating groups based on condition, with preceding analyses showing low statistical power in separated cohorts.

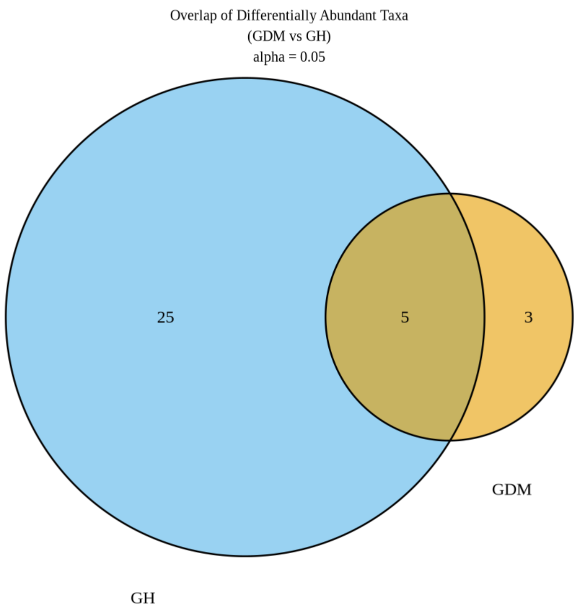
Overlap of Differentially Abundant Taxa
(GDM vs GH)
alpha = 0.05
25
5
3
GDM
GH
